# Immersive interfaces for clinical applications: current status and future perspective

**DOI:** 10.3389/fnbot.2024.1362444

**Published:** 2024-11-27

**Authors:** Naïg Chenais, Arno Görgen

**Affiliations:** ^1^Swiss Center for Design and Health, Nidau, Switzerland; ^2^Department of Ophthalmology, Jules-Gonin Eye Hospital, University of Lausanne, Lausanne, Switzerland; ^3^Swiss Center for Game Design Studies, Institute of Design Research, Academy of the Arts, Bern University of Applied Science, Bern, Switzerland

**Keywords:** immersive technologies, neurodesign, serious games, extended reality, virtual reality, augmented reality, digital education, digital therapeutics

## Abstract

Digital immersive technologies have become increasingly prominent in clinical research and practice, including medical communication and technical education, serious games for health, psychotherapy, and interfaces for neurorehabilitation. The worldwide enthusiasm for digital health and digital therapeutics has prompted the development and testing of numerous applications and interaction methods. Nevertheless, the lack of consistency in the approaches and the peculiarity of the constructed environments contribute to an increasing disparity between the eagerness for new immersive designs and the long-term clinical adoption of these technologies. Several challenges emerge in aligning the different priorities of virtual environment designers and clinicians. This article seeks to examine the utilization and mechanics of medical immersive interfaces based on extended reality and highlight specific design challenges. The transfer of skills from virtual to clinical environments is often confounded by perceptual and attractiveness factors. We argue that a multidisciplinary approach to development and testing, along with a comprehensive acknowledgement of the shared mechanisms that underlie immersive training, are essential for the sustainable integration of extended reality into clinical settings. The present review discusses the application of a multilevel sensory framework to extended reality design, with the aim of developing brain-centered immersive interfaces tailored for therapeutic and educational purposes. Such a framework must include broader design questions, such as the integration of digital technologies into psychosocial care models, clinical validation, and related ethical concerns. We propose that efforts to bridge the virtual gap should include mixed methodologies and neurodesign approaches, integrating user behavioral and physiological feedback into iterative design phases.

## 1 Introduction

Digital immersive technologies have attracted considerable interest in the landscape of biomedical research, due to their versatile range of applications, spanning from patient data visualization, digital Games for Health (GfH) to neurorehabilitation. The use of digital virtual interfaces and augmented reality-assisted technologies have especially gained traction within clinical settings. These technologies are utilized not only to train medical professionals but also for research, education, and patient-centered purposes, including occupational therapy, cognitive, visual, and motor rehabilitation and diagnosis (Yeung et al., 2021). A distinction can be drawn among applications that serve preventive, therapeutic, diagnostic, informative, or educational purposes (Sawyer, 2008). Nevertheless, the primary focus of technology developers tends to revolve around primary therapeutic outcomes and overall usability, but often neglect fundamental plasticity mechanisms, end-user inputs and social context (Mummah et al., 2016; Birckhead et al., 2019).

Biomedical engineers often overlook the intricate user-interface interactions and their long-term interplay with visual and cognitive functions, learning and rehabilitation mechanisms. The chosen technology, intermediate objectives, immersive universes, and communication modalities are more often design choices than evidence-based decisions (Birckhead et al., 2019). There is a limited number of studies addressing the discursive, social construction of immersive interfaces as a novel medium within the field of the social system of medicine. Despite the innovative advancement of various immersive interfaces for diagnosis, education, or rehabilitation purposes, studies are often not designed to effectively compare digital approaches with non-digital methods. Numerous trials investigating the clinical efficiency of immersive interfaces lack sham group, and more comprehensive randomized trials are still in an early assessment phase (Gerber et al., 2017, 2019a,b; Saraiva et al., 2018; Yeung et al., 2021; Jiménez-Rodríguez et al., 2023). Furthermore, the existing comparison studies most fail to demonstrate an advantage of digital over non-digital approaches. Pilot studies including conventional therapy or a placebo control group have demonstrated limited and individually variable advantages of digital methods, with the notable exception of chronic pain alleviation (Maddox et al., 2022) and surgical training (Grantcharov et al., 2004; Lohre et al., 2020; Sadek et al., 2023). Finally, there is a lack of publicly accessible insights into the production processes of such applications, which would make their development traceable and provide insight on detailed design evaluation.

Currently, the success of a given immersive biomedical approach cannot be differentiated from the success of the interaction design choices that have been made (Garrett et al., 2018). Designing for immersive interaction requires consideration of human sensory perception, cognition, and sensorimotor systems and social context. This knowledge has to be carefully integrated into the design process. This applies all the more if the interface is to be used in a clinical setting, as design choices have the potential to impact healthcare processes and patient outcomes negatively (Garzón et al., 2019; Chang et al., 2022). It is today’s challenge to determine which immersive approach and design choices are advisable for which goal and learning context, and to create a general framework of understanding to facilitate further Digital Therapeutics (DTx) development, and design (or neurodesign) brain-centered interfaces (Ahram et al., 2016; Auernhammer et al., 2023).

Despite yet mixed outcomes, the growing number of projects developing and analyzing immersive gaming approaches in the clinics shows the professionalization and institutionalization of the field. The emergence of general interest networks, such as the German “Netzwerk Serious Games und Gamification for Health” signals that the field reaches a stage where tools are moving beyond prototyping. A shared understanding of effective practices is beginning to form, with increased collaboration and dissemination of results. Our objectives with the current review are to contribute to this ongoing process; to further stimulate cross-disciplinary interest and dialogue between the different disciplines involved in that field; and to include social, ethics, and neuroscience perspectives in the emerging voices formalizing future solutions. We propose to specially put in perspective the concepts and recent advancements in immersive technologies, perceptual neuroscience, game design and neurodesign domains.

**Figure 1:**
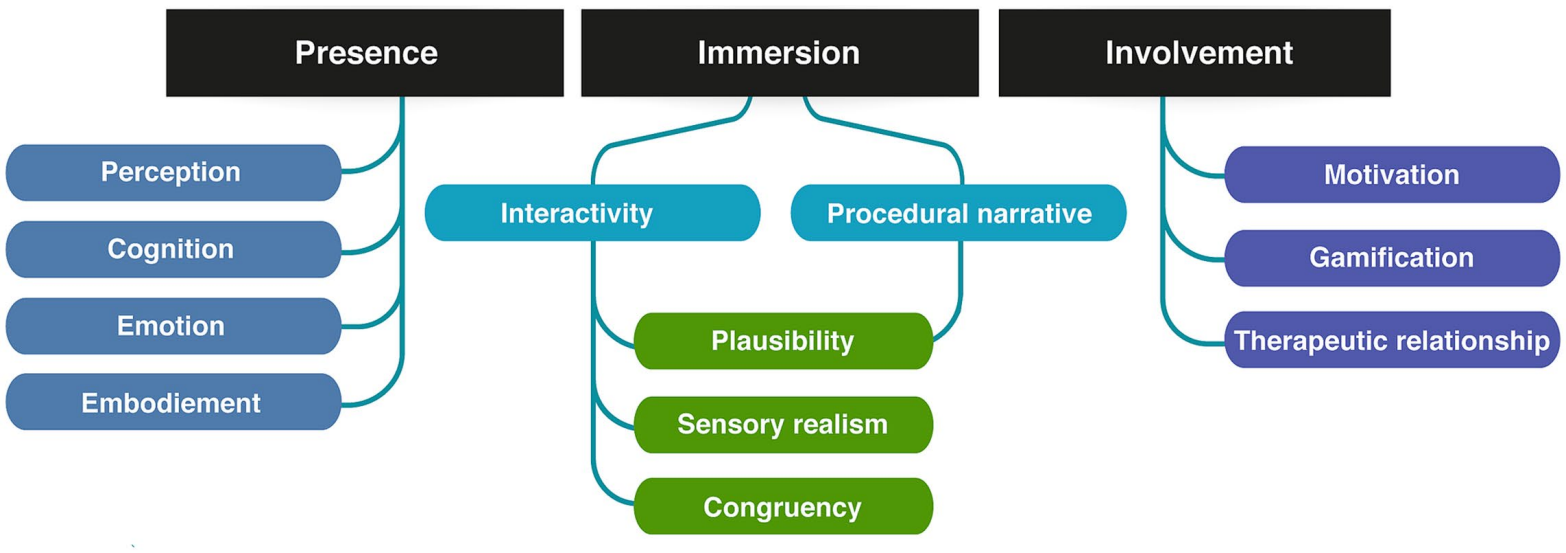
Elements of the positive immersive user experience.

**Figure 2:**
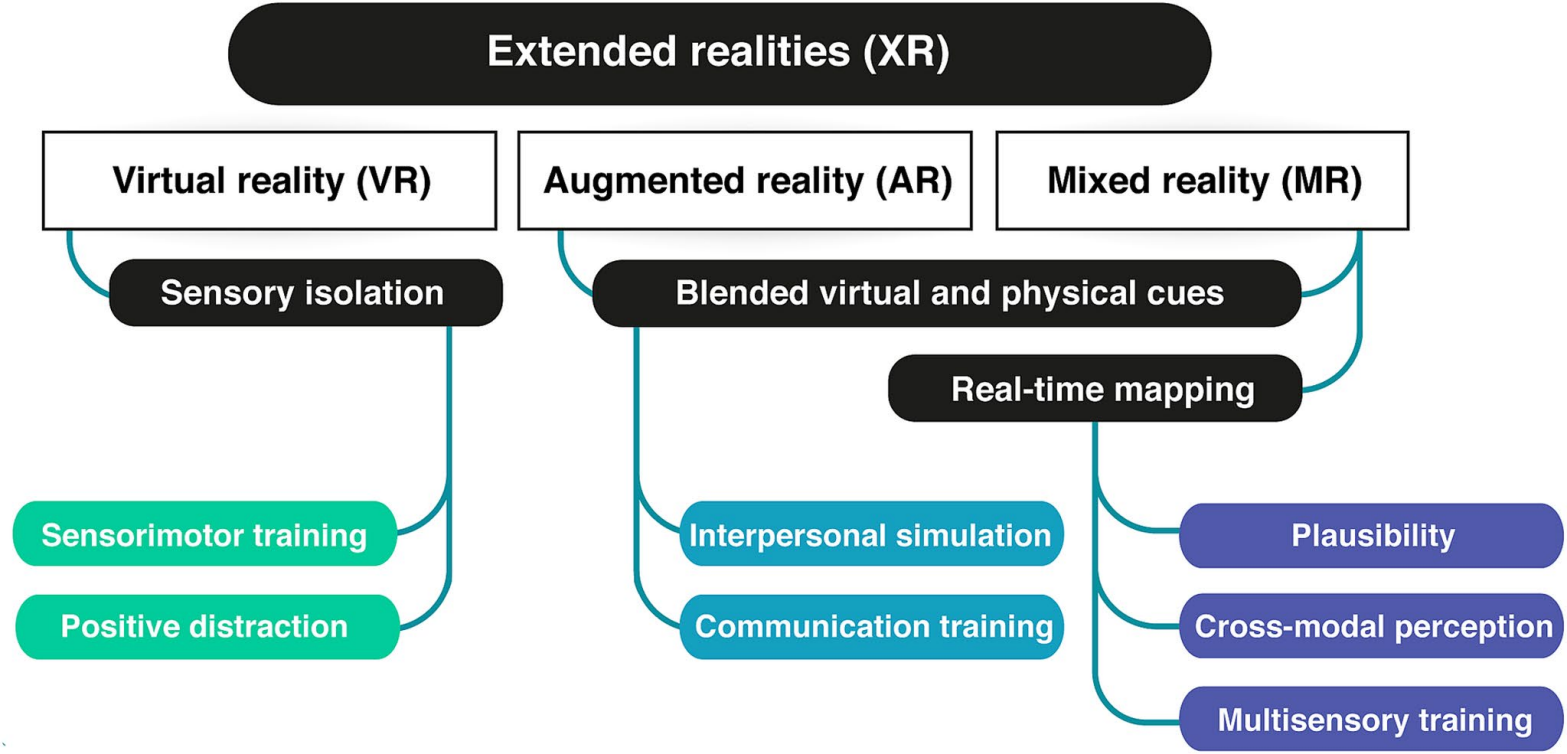
XR technologies and their main contributive advantage for clinical applications.

**Figure 3:**
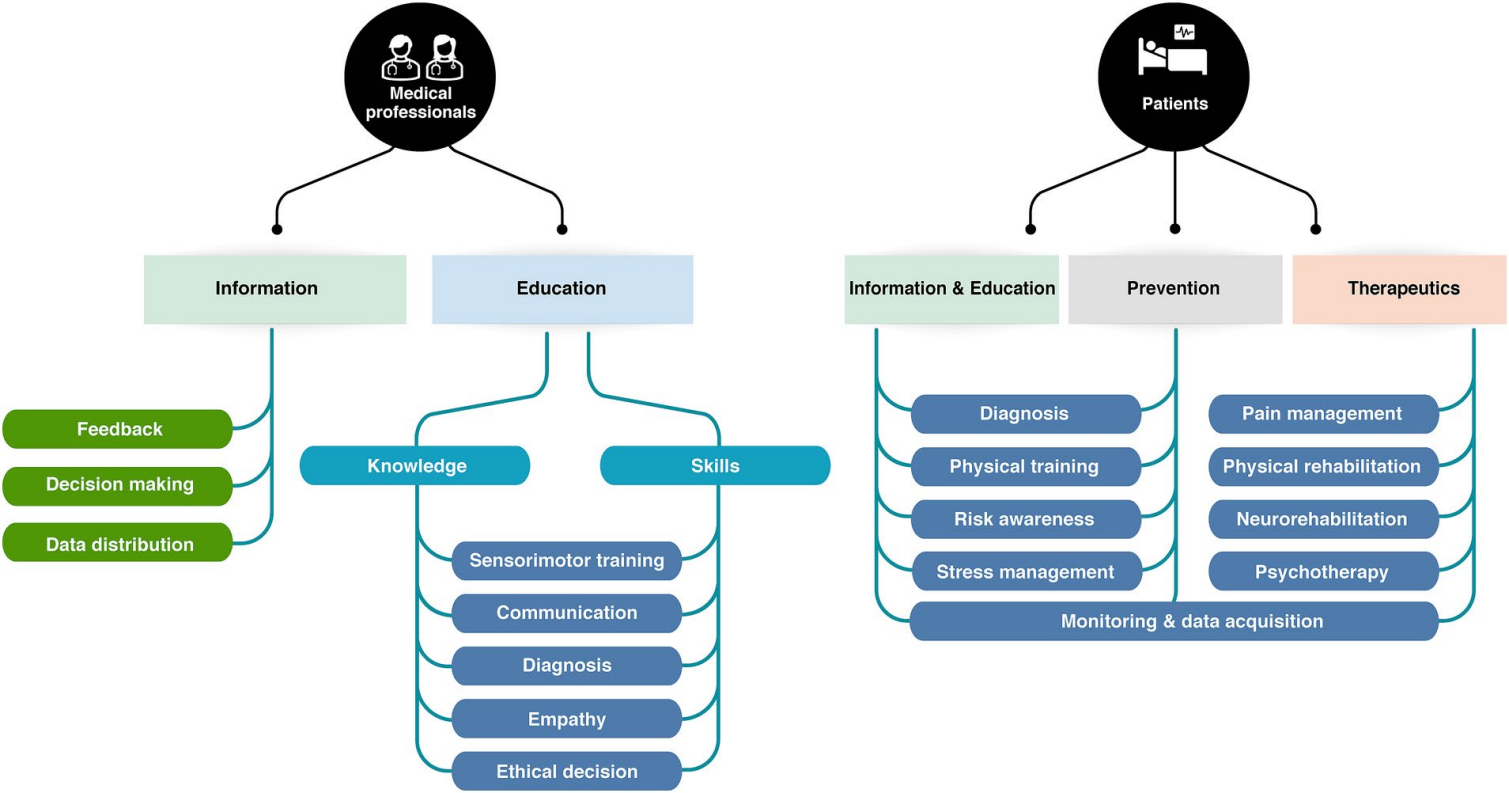
Diversity of applications of XR technologies for patients and healthcare professionals.

**Figure 4:**
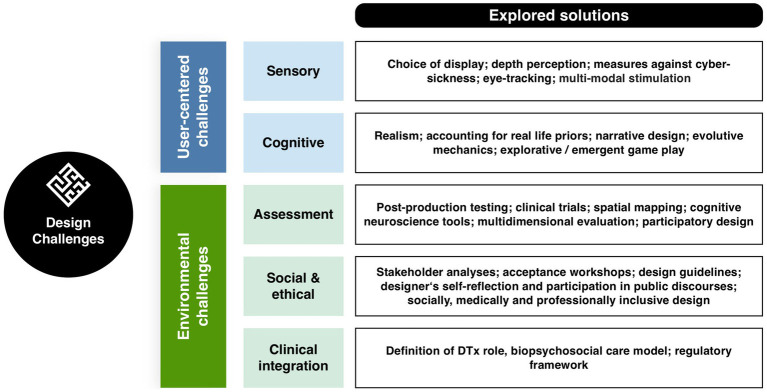
Overview of the design challenges associated with the development of XR interfaces development and their integration into healthcare.

To achieve this, we will first explore the key components and usages of immersive interfaces (2). We will differentiate and define involvement, immersion, and presence as three distinct aspects of the user experience (2.1, Figure 1). Next, we will provide an overview of the technologies in use, including Virtual, Augmented and Mixed realities (2.2, Figure 2). Given the broad range of clinical applications, we will examine how these technologies are currently used (2.3, Figure 3) and identify the primary mechanisms that support this use in practice and favor learning from the perspective of various disciplines (2.3). We will then address the design of actual applications and their challenges (3, Figure 4), focusing on sensory design issues (3.1), cognition and game design (3.2), methods for assessing user progress and behavior (3.3), social (3.4) and regulatory challenges (3.5).

## 2 Immersive interfaces for clinical applications: development and definitions

### 2.1 The immersive experience

Immersive technologies such as Virtual Reality (VR), Augmented Reality (AR), GfH, and other interactive virtual mediums, have been progressively integrating into the realm of healthcare. These technologies, initially created for entertainment purposes, have started to permeate medical practices, and unlocked novel ways to engage the patient, the clinician, and the medical team. The common ground of these technologies is to create a positive user experience (UX) by integrating digital elements into the user’s physical world, thereby enhancing the perceptual and cognitive aspects of user interactions. Yildirim et al. state that three interconnected factors must be ensured for a positive UX: presence, immersion and involvement (Yildirim et al., 2018; see Figure 1).

Presence or the sense of “being there” are much-explored cognitive psychology and consciousness research concepts, often used as subjective metrics to evaluate participants experience in Virtual Environments (VEs) (Riva et al., 2003; Pillai et al., 2013; Grassini and Laumann, 2020; Cypress and Caboral-Stevens, 2022). Presence refers to “a mental state in which an individual feels like they are in an environment other than the one they are physically occupying” (Yildirim et al., 2018). Such mental state is not specifically bound to a type of immersive technology but rather “formed through an interplay of raw (multi-)sensory data and various cognitive processes […] in which attentional factors play a crucial role” (Riva et al., 2003). Sense of presence (SoP) thus describes the minimal emotional and experiential access to another reality—that of the VE for instance. This makes the philosopher de Vignemont describe it as a buffer zone “between the self and the external world […], a place in which objects and events have a unique immediate significance for the subject because they may soon be in contact with [them],” triggering an “illusion of non-mediation” (Lombard and Ditton, 2006; De Vignemont, 2021). Presence therefore relates to the perception of one’s peripersonal space and the likelihood of interaction with the media elements (Riva et al., 2003; De Vignemont, 2021). Precisely because it lies at the interface between perceptual, cognitive and emotional experience, presence is a key component to empower users and reinforce learning, notably in clinical settings. The extent to which users experience SoP in a VE depends on both their involvement and immersion in the environment (Witmer and Singer, 1998).

Involvement can be described as “a mental state in which attentional resources are allocated to the processing of perceptual stimuli rendered in the VE” (Yildirim et al., 2018).,This relates to a much more diffuse aspect of immersive GfH: they are entertaining. Games are designed to generate elevated levels of motivation and engagement in the players, which means that the motivation to use them may be significantly greater in comparison to conventional medical practices (Watters et al., 2006). Yet, GfH and immersive technologies are susceptible to the criticism of being “sugar-coated broccoli,” meaning that the UX differentiates from what GfH promise to offer, namely a gaming experience that can compete with commercial games (Baranowski et al., 2016). In that way, the user involvement depends on the immersion and presence brought by both the interaction design and the narrative of the GfH. From a UX perspective, the product of involvement and immersion has been conceptualized as flow, i.e., the absorption in an activity, during which irrelevant thoughts and perceptions are being screened out (Jennett et al., 2008). Flow is associated with the following components: “clear goals; high degree of concentration; a loss of the feeling of self-consciousness (sense of serenity); distorted sense of time; direct and immediate feedback; balance between ability level and challenge; sense of personal control; intrinsically rewarding.”

Finally, immersion is a “a psychological construct concerned with the extent to which individuals feel encapsulated by the VE and perceive themselves as an integrated component of the environment” (Yildirim et al., 2018). The two major components of immersion are, on the one hand, interactivity, i.e., the user’s possibility to meaningfully influence the media content and alter it within the framework and possibility spaces granted by the media (Bodi and Thon, 2020); and on the other hand, procedural narratives (Bogost, 2010). Such narratives unfold primarily in the form of systemic processes: users must do action x to get result y. Ultimately, interactivity and narrative allow for immersion from a story building perspective and make GfH suitable for use in a medical context. Translating this definition into a visual communication and digital implementation perspective, immersion becomes the sense that digital virtual objects belong to the real world (Stevens, 2021). This implies the viewer to integrate together perceptions from the digital and physical worlds, and to interact with digital or physical elements equivalently. This equivalence is a key factor to the generalization of learning when using serious games. The crucial aspect in this context is the perception of immersion as a bottom-up phenomenon. Donghee Shin highlights that “[t]he meaning of immersion depends on the users’ idiosyncrasies, and the influence of immersion substantially depends on the users’ contexts such as their pre-existing conditions and personal traits. […] Immersion is a form of awareness in the eye of the beholder, and its degree reflects the intensity of the users’ cognitive, emotional, and sensory connections to both the content and form of the product” (Shin, 2019).

The various forms of existing extended reality technologies reflect not only the available technological advances, but also users’ presumed needs in term of involvement, immersion and presence. Focus is either made on physical immersion (“from every angle”), sensory immersion (“from all the senses”), involvement (“that do not allow distracting information”) or presence (“that does not provide diverging information”).

### 2.2 Current immersive technologies

There are several types of immersive technologies that differ in their degree of immersion and the way they blend virtual and physical elements (Milgram and Kishino, 1994; see Figure 2). Extended reality (XR) is an overarching term that refers to hybridization of virtual and physical elements into a more complex immersive perceptual space. XR comprises virtual reality (VR), augmented reality (AR), and mixed reality (MR). All XR modalities are explored for health and clinical applications, though VR has been the format preferred for GfH development in the past decade.

VR immerses users in computer-generated or digitally-presented environments. Presentation is typically achieved through head-mounted displays (HMDs) or computer-assisted virtual environments (CAVEs). VR typically isolates users from their physical surroundings. This isolation primarily concerns visual and auditory systems, but efforts are made to create congruent haptics (Jerald, 2015; Stevens, 2021). This multisensory isolation perspective hallmarks VR for multiple clinical applications, particularly in sensory training, but it has been investigated for its potential to serve as a positive distraction during medical procedures (Dumoulin et al., 2019; Faruki et al., 2022) or to create calming environments (Gerber et al., 2017, 2019b).

AR overlays virtual content onto the real-world environment, either directly projecting cues onto the physical world, via mobile digital interface, or wearable devices. The added information can be visual cues enhancers, text, or local distortions, audio stimuli and audio-description of visual cues. AR can be implemented on personal mobile devices or through projection mapping: visual information can be projected to 360° onto multiple physical displays, such as circular screens or projection floors, eventually paired with spatial audio rendering. Projection mapping limits multisensory immersion but promotes interpersonal interaction and communication. For this reason, it has been used to explore medical communication (Aliwi et al., 2023; Song et al., 2023), medical architectural design (Afzali et al., 2023) and to facilitate social communication between children with auditory impairment, cognitive impairment, or autism spectrum disorders (ASD) and their relatives (Richard et al., 2007; Liu et al., 2017; Sahin et al., 2018; Tenesaca et al., 2019). In clinical contexts, AR can also be implemented on optical see-through mode (OST). The user perceives unaltered real-world environment through a see-through device, typically goggles, and the supplementary digital content is directly projected onto the retina through the lens. OST is key to visuomotor training and ophthalmology applications as it allows the user to use their normal head and eyes movements to scan the visual scene, what facilitates the processing of visual inputs and the process of learning to use the interface (Hafed et al., 2016; Thorn et al., 2022).

MR combines elements of both VR and AR, mapping virtual and real-world environments together, and foremost allowing user to interact with both worlds. MR differs by its dynamic multidimensional technology and flexibility. MR relies on real time scanning of the user’s visual space with a 3D laser technology, usually integrated on the image acquiring device: a virtual mesh of the real world is built on which virtual objects to be manipulated and interacted with. The visual environment is continuously adjusted and aligned with the user’s visual field, its motion speed, and the mesh adapts to create depth and proportion perception through occlusion, shadows, aerial or linear perspective. The latter two features are key for establishing plausibility (Jerald, 2015). Some MR models also incorporate real world mapping, such as GPS coordinates or known floor plans (Jerald, 2015; Syahputra et al., 2020; Verma et al., 2020), substituting visual scan to positional and kinematic information.

### 2.3 Users and applications

In clinical health, immersive technologies have primarily three categories of users: medical professionals, patients or potential patients, and healthcare planners. Interfaces for medical staff training are designed for multidirectional thinking, enabling the retrieval of diverse health data and parameters, processing of knowledge, generation of decisions in the form of a coordinated care plan or medical actions, and provision of feedback (see Figure 3). Most interfaces are designed to provide information related to diagnosis (such as rehabilitation interfaces), to train medical communication skills (especially in nursing and psychotherapy fields), or to train technical sensorimotor skills (through anatomical simulation) (Hussey, 2021; Tene et al., 2024). Conversely, most interfaces geared for patients are designed for goal-oriented thinking; and focus on transformational outcomes, i.e., lasting changes in the user behavior, knowledge, priors, cognitive, emotional or sensorimotor skills (see Figure 3). XR interfaces have been developed for prevention and awareness, relaxation, and DTx—especially in neurorehabilitation and psychiatry fields. Finally, interfaces used by health planners are intended to provide insights, observational feedback, and design guidelines. This field of application is still emerging to date, but research efforts are made to develop XR tools for evidence-based healthcare environment design (Bianco et al., 2016; Aoyama and Aflatoony, 2020; Hwang and Shim, 2021; Afzali et al., 2023).

A substantial number of immersive interfaces developed over the past 15 years are geared towards health professional education. Immersive interfaces have been developed to train various core aspects of clinical care: anatomy knowledge, anamnesis, diagnosis, interpersonal communication during care, ethical decision making, medical acts, and surgical procedures (Grantcharov et al., 2004; Bhoopathi and Sheoran, 2006; Garzón et al., 2019; Lohre et al., 2020; Chang et al., 2022; Sadek et al., 2023). XR environments are acknowledged valid methods for clinical education and training, but evidence suggests that they should rather be envisioned as an auxiliary consolidating medium for related learning (Kyaw et al., 2019; Kim and Ahn, 2021; Sadek et al., 2023). Kyaw and Tudor Cal state in their meta-analysis that “only low-quality evidence show(s) that digital education is as effective as traditional learning in medical students’ communication skills training. Blended digital education appears to be at least as effective as, and potentially more effective than, traditional learning for developing communication skills and knowledge” (Kyaw et al., 2019). For anatomy learning and specific technical and surgical skills acquisition, XR immersive learning generates a more nuanced picture. Students perform equally when taught with immersive or traditional methods, but acquire skills significantly faster with virtual immersive environments, (Jordan et al., 2001; Eichenberg, 2012; Lohre et al., 2020). For rare or high-risk neurological or ophthalmic surgical procedures, XR environments are often the only way trainees can have hands-on realistic training (Butt et al., 2018; Muñoz et al., 2022). Finally, XR training can be exerted without ethical considerations of the patients’ consent to be an *in-vivo* or post-mortem exercise subject. This suggest that the main interest of medical educational immersive interfaces do not reside in clinical efficacy, but in resources. They might offer a fast, reliable, mistake-allowing training to students and reduce the pressure on training time, availabilities, and ethics. Importantly, VE learning alone is not the preferred learning tool for medical students, but still needs observation and mentorship as complimentary didactical approaches (Engum et al., 2003; Williams, 2019; Vizcaya-Moreno and Pérez-Cañaveras, 2020). Last, the above-mentioned studies all focus on knowledge and skill retrieval from a user perspective. Patient-related outcomes, as well as adverse effects, and cost-effectiveness of immersive digital education are still case-specific open questions.

Immersive technologies and GfH intended for (potential) patients focus on the transformational potential of the user’s experience, and on the real-word transfer and persistence of the acquired skills. All GfH can be described as transformational, but the intricacy between entertainment and learning is more pronounced when game mechanics is part of therapeutic process (Culyba, 2018). XR technologies for patients most often fall into two main categories: DTx, and preventive applications. DTx are evidence-based digital products aiming at preventing, managing, or treating treat health conditions (Digital Therapeutics Alliance, 2023), and lie within the broader category of digital medicine (Wang et al., 2023). In this article, the term DTx is used to denote prescription-only interventions that have been developed with clinical evidence and are intended for the treatment of specific medical conditions, particularly major chronic diseases.

Preventive applications are developed for either general or risk-specific audiences, sometimes in collaboration with entertainment studios. They aim at transforming the user cognitive priors and habits on a health topic, through medical information delivery or training. Examples of preventive GfH include the niche application Playforward - developed to reduce HIV exposure in at-risk teen populations (Fiellin et al., 2016), and the more popular WiiFit exergames (Tripette et al., 2017). In such GfH, participatory methods are made possible by two key factors: a healthy target group—limiting the need of medical supervision and multidisciplinary safety evaluation, while allowing a large dataset acquisition-; and overly broad expected outcomes—allowing exploratory methods to evaluate potential clinical benefits. In the hospital settings, preventive applications aim at patient relaxation and distraction, helping patients to manage pain and anxiety during medical procedures. Research efforts have notably focused on stress reduction in the emergency department and intensive care unit (Gerber et al., 2017, 2019a; Dumoulin et al., 2019; Hill et al., 2022; Vlake et al., 2022). Preventive applications only represent 4.4% of the clinical trials in digital medicine (Wang et al., 2023). Commercial interfaces are often evaluated *post hoc* by independent research groups (Li et al., 2011; Bonnechère et al., 2016; Tripette et al., 2017).

XR-based DTx focuses on chronic neurological and psychiatric diseases, that require continuous interaction. They indirectly interface with patient nervous system through behavioral intervention, to favor plastic changes in sensorimotor and cognitive loops, and allow modification or acquisition of new behavioral, sensory, and motor skills. Their mechanisms do not essentially differ from that of educative immersive interfaces, however, their regulatory framework do. DTx represents the majority (65%) of clinical trials conducted in the digital medicine sector worldwide and are usually classified as class II medical devices (Wang et al., 2023; Watson et al., 2023).

Neurorehabilitation is a major application field of immersive DTx: specific interfaces have notably been developed for motor (Vinolo Gil et al., 2021; Chen et al., 2022; Gorman and Gustafsson, 2022; Yang Z-Q et al., 2022), cognitive (Richard et al., 2007; Laver et al., 2017; Miskowiak et al., 2022), speech (Da Silva et al., 2015; Xu et al., 2020; Bu et al., 2022) and visual rehabilitation (Li et al., 2011; Ghali et al., 2012; Boon et al., 2017; Saraiva et al., 2018; Jiménez-Rodríguez et al., 2023). A major advantage of XR training is to provide tailored and realistic sensory feedback on motor actions, which is essential for sensorimotor training. Continuous feedback can be visually delivered through avatar posture and gestures; on–off targeting accuracy feedback can be provided visually or haptically through controllers; and proprioceptive feedback can eventually be provided through flooring force plates and postural platform. The early development of preventive balance exergames for general audience paved the way for DTx applications. But these commercial exergames are often used with little adaptation for clinical monitoring and disabled population (Chao et al., 2015; Bonnechère et al., 2016). Further research is needed to understand how various feedback modality and types of visual feedback and point of views contribute to sensorimotor reeducation, and which plastic mechanisms can be exploited for tailored XR rehabilitation interfaces. A notable advantage of XR therapy is the opportunity to pair it with robot-assistance for more reliable quantitative measures of patient performance and online tasks adjustment during sessions (Vidrios-Serrano et al., 2015). XR technology is also exploited for sensory substitution training in the visually impaired (Dragos Bogdan Moldoveanu et al., 2017).

The second major clinical application for immersive DTx is psychotherapy. XR technology is a first-class tool to design behavioral interventions, particularly for exposure therapy, exposure with response prevention, and cognitive-behavioral therapy (Grochowska et al., 2019), notably in contexts of Post-Traumatic Stress Disorders (PTSD) (Rothbaum and Schwartz, 2002; Kothgassner et al., 2019; Eshuis et al., 2021), eating disorders’ body disturbance (Ferrer-García and Gutiérrez-Maldonado, 2012; Riva et al., 2021; Behrens et al., 2022), phobias and social anxiety (Schoneveld et al., 2018; Boeldt et al., 2019; Wechsler et al., 2019). It can help the therapist understand the fictive situation, and assess patient behavior, addressing significant shortcomings of existing therapies (Boeldt et al., 2019). AR behavioral interventions have also been specifically developed for collaborative therapies in children and adults with ASD. Contrary to VR exposure therapy, this application merges real-life situations and virtual information, with the objective of assisting and training social cognition skills in ASD users (Da Silva et al., 2015; Liu et al., 2017; Sahin et al., 2018). Last, VR GfH has been developed for avatar therapy in schizophrenic patients (Mayor, 2017; Ward et al., 2020; Verma et al., 2020).

To date, the majority of immersive interfaces for neurorehabilitation and psychotherapy uses VR, with the notable exception of sensory substitution and sensory pain applications, whose intrinsic mechanisms rely on a combination of virtual and real-life stimulations.

### 2.4 Exploited mechanisms

Immersive applications and GfH are extremely tailored to their user group and their intended use. To implement this focus effectively, a series of game and UX mechanisms should be implemented in a targeted manner.

#### 2.4.1 Embodiment and presence

Presence and SoP are a core mechanism that mediates positive immersive UX, XR-trained skills acquisition, transfer, and retention. SoP refers to spatial and plausibility illusions, also referred to as illusion of non-mediation in XR (Slater, 2009; Slater et al., 2010a). This misperception has been foreseen as multimodal perceptual illusion of self’s body and peripersonal space (Riva et al., 2003; De Vignemont, 2021). Embodiment and SoP are intricated in XR, although only recently conceptualized together (De Vignemont, 2011; Forster et al., 2022). Embodiment describes the process of integrating external bodily entities (as an avatar) into self-body representation – in a similar way external entities can be integrated into peripersonal space representation. In VR in particular, SoP mostly depends on embodiment users have to embody the virtual body to feel present enough in the VE. This mechanism is directly exploited in digital therapy to alleviate phantom limb pain (Hunter, 2003; Foell et al., 2014; Romano et al., 2016; Osumi et al., 2017), and avatar therapy for schizophrenic and psychotic patients (Carter et al., 2017; Mayor, 2017; Ward et al., 2020). Interfacing with VE and avatar can teach latter patients to question the boundaries between external and internal percepts, or alternatively provides them with a virtual object to which they attribute the hallucinated percepts (Ward et al., 2020).

Embodiment may be limited by either bottom-up (Slater et al., 2010b), or top-down factors, such as virtual body characteristics and point of view (Ehrsson et al., 2004; Tsakiris, 2010; Romano et al., 2016), and its plausible connection to the user body. Third-person point of view, despite facilitating visual design, hampers SoP (Jerald, 2015). However, preferred avatar modalities for given applications are still unclear. The term “avatar” describes a user’s representative in the VE, as an immersion medium, “persona” refers to the product of such immersion and embodiment, i.e., the self-perception of the user in the virtual or extended environment. A recent study showed that an unrealistic but highly controllable first person-perspective avatar allowed higher embodiment than realistic but less flexible avatar (Fribourg et al., 2020). Third-person point of view personas can also be especially important for psychotherapy applications, allowing a trade-off between high emotional engagement and limited SoP (Ward et al., 2020). Personas are also a valuable tool for co-design process and empathy generation (Ventura et al., 2020).

The major contributing factors to embodiment and SoP are sensory realism and crossmodal perception (Lopez et al., 2008; De Vignemont, 2011; Blanke, 2012). Most VR and AR systems are designed to provide a virtual stimulus to the dominant visual sensory modality, unless they specifically target visually impaired audience, or are oriented towards specific haptic uses (Ghali et al., 2012; Borja et al., 2018). However, the integration of multisensory inputs, and the congruence of various cues are key to self-perception, embodiment, and SoP (Lopez et al., 2008; Blanke, 2012; Cao et al., 2019). A major challenge of XR is to integrate and provide multiple sensory modalities together. One specific challenge is integrating haptics with immersive visuals: few haptic devices today are wireless, what hampers the free movement of the user in the XR environment. A proposed solution is to integrate passive haptic feedback and control into augmented floors: such passive haptics have a positive impact on SoP and allow multiuser interaction (Law et al., 2008; Goncalves et al., 2020), but has yet not been implemented for clinical applications.

Social factors also influence embodiment and SoP. Openness to virtual immersion, medical education and occupation, confidence in medical preparation, and previous exposure to the simulated situation influence SoP during medical XR simulation (Paquay et al., 2022). SoP is also influenced by social factors, such as gender (Grassini et al., 2020). Higher SoP was reported in women during medical XR training (Grassini et al., 2020), and possibly relate to their better learning performance (Yang et al., 2016). Conversely, the lower SoP in man students may be associated to increased confidence in educational preparation and medical proficiency (Blanch et al., 2008; Flyckt et al., 2017; Vajapey et al., 2020).

#### 2.4.2 Procedural learning

Procedural learning pertains to the acquisition of complex and adaptive motor or cognitive skills, i.e., skills that involve constant decision and action rules update. Procedural learning is required for sensorimotor, cognitive, emotional and social competencies acquisition during clinical education, physical or cognitive therapies. According to the adaptive control of thought model (Anderson, 1982, 2000), training pattern plays a significant role in automatizing the learnt procedural skills (Miyake et al., 2000; Shahar and Meiran, 2015). Training through simulation has shown satisfying efficiency for medical procedural training (Nestel et al., 2011), and is routinely used in medical education for developing technical motor skills, as well as for learning analysis pathways and communication methods learning.

XR technology offers several advantages for simulation-based procedural learning. First, XR offers the possibility to overlay the simulated situation with explicit information. It enables to combine exploratory learning with on request explicit information display. Indeed, the two main methods to acquire procedural skills are instruction-based training and exploration-based training. Exploration-based training, also referred to as active learning, facilitates the induction of abstract representations and procedural knowledge by employing analogical reasoning (Kamouri et al., 1986). Exploration-based learning is centered on the active engagement of the user, and both gamification and immersion technologies are privileged tools to achieve so. Second, procedural learning requires constant shift between environmental action cues in a goal-directed manner. Working memory, information update, and cognitive inhibition are key to initiate procedural learning (Miyake et al., 2000; Shahar and Meiran, 2015). Real-life environment holds a host of action cues; but in VEs, the dimensionality of available cues is intentionally reduced and carefully selected by design. This reduced dimensionality might facilitate the information sorting process and reduce the cognitive load during the cognitive phase, which can explain the fastening of procedural learning in VR-based surgery training or communication skills training.

Meta-reviews point out that the 3D design of a XR interface is a major predictor of procedural learning performance (Garzón et al., 2019; Chang et al., 2022). Importantly, procedural learning abilities can be altered in populations using XR interfaces for cognitive therapies training, such as neurodegenerative patients (Soliveri et al., 1992; Muslimovic et al., 2007; Clark and Lum, 2017), but also users with ASDs, though diverging evidence (Mostofsky et al., 2000; Clark and Lum, 2017; Boucher and Anns, 2018).

#### 2.4.3 Perceptual learning

Perceptual learning refers to a lasting alteration in perception resulting from experience. It can be categorized as a form of implicit or procedural learning involving sensory systems. Perceptual learning differs from sensitization and habituation, as it leads to permanent improvements in perception and perceptual thresholds. Importantly, it can occur independently of both attention to the stimulus and conscious perception (Watanabe et al., 2001). Perceptual learning underlies active sensory rehabilitation trainings, such as low vision or sensory substitution trainings, but also education trainings related to discrimination tasks—such as medical images interpretation (Seitz, 2017; Serrada et al., 2019). In a clinical context, the process may entail distinguishing between simple and complex classes of stimuli, such as the histology of various tissues for surgical training, or distinct types of auditory information during low-vision training (Fahle, 2009). In adults, perceptual learning depends on prolonged and repeated exposure, strength of exposure, and various additional factors, including attention, reinforcement, and interactions of multiple sensory systems (Seitz, 2017). These factors are actively investigated into perceptual learning research field, but receive less attention when designing applied XR and GfH interfaces. Consequently, there is currently no strong evidence regarding the effectiveness of XR-based training on perceptual thresholds in sensory-impaired patients (Polat, 2009; Serrada et al., 2019).

Due to the visually dominant nature of most XR outputs, visual learning is the most prone to occur with XR training. Perceptual learning can lead to very fast improvements of discrimination performance in visual tasks. Yet, perceptual improvement is often highly specific for the trained task, stimulus orientation and position in the visual field (Fahle, 2004, 2009), what limits the generalization of the perceptual improvements to other tasks. The positive influence of top-down factors such as feedback and attentional control (Ahissar and Hochstein, 1993; Herzog and Fahle, 1997, 1998; Fahle, 2004; Seitz, 2017) open opportunities for better perceptual training design.

#### 2.4.4 Positive reinforcement learning

Positive reinforcement is a common feedback-driven mechanism for multiple forms of procedural, associative and perceptual learning, including purely implicit sensorimotor learning (Law and Gold, 2009; Wächter et al., 2009). Reinforcement learning is an adaptive process in which the user’s previous experiences are used to predict the outcome of possible action and make a choice accordingly. Action choices can be made at various levels: among alternative single motor outcomes, among different complex actions or objects selection, or among different interaction strategies. Reward-based learning is an essential element of gamification in GfH and immersive interfaces. Rewarding outcomes can be explicitly included in the game mechanics; visual, auditory, or haptic feedback of task completion or accuracy can also be considered as a rewarding element. In educational and DTx, the rewarding elements can also arise from patient-therapist verbal interactions, and from other users’ interaction in collaborative training. Reward type, timing, and predictability matter for serious games impact. Reward-based game mechanics based on badges and trophies was found to have greater positive influence on learning than points scoring and meaningful educational messages delivery (Whittaker et al., 2021). Granting rewards after an unpredictable number of correct trials and adjusting rewards to the user individual occurrence preference were associated with higher enjoyment, improved learning performance, and longer durations of gameplay (Nagle et al., 2014). Comprehensive and generalizable studies on the use of serious games and DTx rewards-based reinforcement are still required.

However, educational technologies based solely on positive reinforcement learning raise several concerns, not unlike those raised by Skinner’s teaching machines (Skinner, 1961). Skinner’s radical behaviorism was a tempting education research program until the beginnings of the cognitive science revolution in the 60s. It postulated that behaviors could be fully understood as overt actions and motor outcomes from physical environmental stimuli (Abrahamsen and Bechtel, 2012). This reductive approach is rooted in experimental approaches of operant conditioning and has long been criticized as oversimplifying human cognition (Cranmore, 2022). Behaviorism survived in several psychological approaches and biomedical fields, such as behavior therapy (notably for autistic and schizophrenic patients, at the origin of today’s XR applications) (Lovaas and Newson, 1976; Stahl and Leitenberg, 1976; Lovaas and Smith, 1988). While a reductionist approach might be relevant to model learning from a fundamental perspective, it is questionable to apply such framework for educational technology. XR applications are still based on experimental approaches, and clinically validated with assessable behavioral or motor outcomes. These plays a crucial role in understanding behavior and training success, particularly in tasks like surgical gesture training or physical rehabilitation. Yet, higher cognitive and emotional processes cannot be neglected in the numerous applications involving therapeutic relationship building.

#### 2.4.5 Symbolic enactment

In transformational XR interfaces with behavioral, psychological, empathy, social communication frameworks, the gaming and immersive aspects can be seen as forms of symbolic enactment. Symbolic enactment is a powerful tool for personal transformation within digital games (Rusch and Phelps, 2020), akin to its role in experimental psychotherapy that incorporates drama, role play, and user active and spontaneous performance (Moreno, 1987; Rusch and Phelps, 2020). Symbolic actions and metaphors in games, performance, and simulations are thought to transcend abstract representation (Thompson et al., 2009; Rusch, 2017). D.C Rusch postulates that gaming metaphors and iconic symbols convey intangible aspects of human experience, constituting a shortcut to experience complex abstract concepts enactment (Rusch, 2017). In immersive interfaces, the experimental metaphor and SoP converge, resulting in a more potent symbolic and enactive experience. Metaphor design holds particular significance in XR interfaces developed for clinical psychotherapy, particularly in the context of exposure therapies for PSTD, eating disorders (Boeldt et al., 2019; Eshuis et al., 2021; Herz, 2021; Behrens et al., 2022), or social immersive training (Liu et al., 2017; Sahin et al., 2018). However, the benefits of metaphorical exposure and virtual immersive exposure—yet perceived as experienced are difficult to distinguish in XR.

In this first part, we have shown how immersive technologies as VR, AR and MR are increasingly used in healthcare, particularly for clinical education, rehabilitation, and therapeutic interventions, and how they can directly or indirectly mediate the medical practices and exchanges between patients and health professionals. These technologies enhance UX by combining virtual and physical artifacts and actions. VR is the most commonly applied technology in serious GfH and for behavioral or cognitive therapy. AR and MR overlays digital elements onto the real world, and are offer additional possibilities for interactive and sensorimotor trainings. These technologies relying on three key factors for users to emotionally and experientially access a VE: presence, involvement, and immersion. Their efficacy for clinical training takes advantage of several learning mechanisms, that could be further addressed by designers. A core element is SoP, influenced by embodiment, where users integrate avatars into their self-representation. Procedural learning in XR benefits from reduced sensory overload and targeted cues, and supports development of complex skill in medical education. Positive reinforcement mechanisms are central to gamification in XR. Lastly, symbolic enactment is particularly key in psychotherapy applications. The interaction between users and the VE are multidimensional: from early sensory processing to complex sensorimotor, emotional and behavioral responses, until transformation of user representations. In the following section, we will analyze these interactions of increasing complexity, the theoretical framework and technologies that make it possible to design them. Design challenges and discussed solutions are summarized in Figure 4.

## 3 Human-computer interaction design

### 3.1 Sensory design challenges

The effectiveness of immersive interfaces in training, rehabilitation, or education hinges on their ability to interact with human senses, cognition, and to generate motor or cognitive actions. To exploit the full potential of recent technological innovation in XR, a deeper understanding of user interaction mechanisms is imperative for maximizing therapeutic outcomes. This observation led to several efforts to integrate cognitive psychology education into developers and computer scientists training (Jerald, 2015; Hodent, 2017). Most XR developers focus on spatial visual and auditory stimulation as fundamental human factors (LaViola et al., 2017). Indeed, space perception is a fundamental prerequisite for immersion and interactivity.

#### 3.1.1 Visual simulation

Visual displays are by far the predominant display devices utilized in XR technologies. Various projection techniques include HMD, tabletop, single screen display, surround screen, multiscreen, or arbitrary surface display. Single-screen displays—conventional monitors, smartphone, or tablet display—are commonly used in clinical XR applications, including GfH, diagnosis and information applications. Surround screens allow users to rely on their peripheral vision, and to move freely within the VE. Such advantages can be essential for biomechanical tracking, visual behavior tracking, and communication studies. Nevertheless, the encoding of depth encoding and 3D objects manipulations in such environments is often inaccurate.

HMDs can either display virtual objects on a head-worn screen or project images directly on the user’s retina (Kollin and Tidwell, 1995). In such virtual retinal display systems, patterned monochromatic light beams are projected through a goggle-mounted OST system. In DTx, virtual retinal displays are predominantly envisioned for ophthalmic applications, in combination with retinal prosthesis or vision restoration interventions (Palanker et al., 2005; Bloch and Da Cruz, 2019; Muqit et al., 2020; Chenais et al., 2021). HMDs can achieve a finer and more naturalistic stereopsis control: stereopsis is directly achieved by the simultaneous projection of one image per eye. However, the tradeoff between constant focal depths and different virtual depths can cause accommodation and vergence conflicts, leading to eye strain and discomfort. Consequently, HMD is not the preferred display source for clinical ophthalmologic applications: diagnosis and visual training commonly rely on non-digital or screen-based digital displays; though multiple VR initiatives are emerging to facilitate diagnosis (Moon et al., 2021; Rajavi et al., 2021; Vicat, 2021; Ma et al., 2022). Comparative advantages of screen-based MR and AR have not been investigated to our knowledge.

In HMDs, integrated eye tracking allows to adjust projected images or virtual elements to a fixed retinal location. The virtual retinal display can increase SoP, reduce cyber-sickness, accommodation, and vergence issues (Jerald, 2015). Integrated adjusting lenses and micro-deformable optics have also been proposed to dynamically adjust the focal plane, and limit vergence and accommodation issues (Love et al., 2009; Zhan et al., 2020; Zhou et al., 2021). These technologies allow users to use their natural accommodative response for depth perception and are promising tools to further develop ophthalmic training applications. However, users suffering from amblyopia or other conditions affecting depth perception cannot perceive 3D effects (Knopf et al., 2017), and compensatory design or evolutive design throughout the visual training protocol must be considered. Neck muscular fatigue and discomfort resulting from prolonged HMD use must also be considered: it is not suitable for physically vulnerable patients, claustrophobic or dement patients, nor for prolonged surgery training.

#### 3.1.2 Auditory cues and audio-visual integration

In the untrained healthy brain, converging information from auditory, visual, and sensory cortices are integrated together to form meaningful multimodal percepts (Marks, 1978; Blattner and Glinert, 1996; Graziano, 2001; Zmigrod and Hommel, 2013). Yet, the different sensory modalities are not equal in VE information integration. Vision dominates auditory and haptic sensory modalities in numerous experimental settings (Colavita, 1974; Blattner and Glinert, 1996; Spence et al., 2012; Bruns, 2019), including virtual reality multisensory display (Gonzalez-Franco et al., 2017). However, visual dominance disappears in a visuo-audio-haptics sensory combinations, and auditory stimulation can be critical to achieve a balanced multimodal information processing and limit the user dependency on visual display (Hecht and Reiner, 2009).

Furthermore, sensory dominance is modified by specific sensorimotor skills acquisition and perceptual training (Colavita, 1974; Powers et al., 2009); and it is altered in specific user groups: patients with neurodegenerative diseases (Murray et al., 2018), motor or sensory disabilities, children with ASD (Hermelin and O’Connor, 1964; Feldman et al., 2018; Ostrolenk et al., 2019). This makes audio-visual integration testing in VE an interesting tool for diagnosis, and a necessary design question. Spatial and timing congruency of stimuli are necessary to audio-visual multimodal integration (Teder-Sälejärvi et al., 2005; Bruns, 2019). In VR, multimodal stimulation, either combining auditory and visual stimulation, or auditory, tactile, and visual stimulation, can decrease the cognitive load of users (Marucci et al., 2021). Audio-visual integration has been identified as promoting embodiment in VEs, and surround auditory stimulation was found to be the preferred display modality to elicit presence neural correlates (Langiulli et al., 2023).

The technological challenge for open VEs and multiple-users VEs is to provide surround ambient sound that dynamically matches with the user spatial location and can serve as a multidimensional information cue (Raghuvanshi and Lin, 2007; Kapralos et al., 2008; Verron et al., 2010; Mehra et al., 2015; Yang J. et al., 2022; Liang et al., 2023).

#### 3.1.3 Olfactive cues

Inclusion of olfactory stimulation has been for long envisioned to develop fully immersive multisensory experience (LaViola et al., 2017). Olfactory stimulation has been identified as a potential contributor to the efficient recall of memories in PSTD exposure therapy (Herz, 2021). The delivery of olfactory cues for medical diagnosis training in humans and animals has been investigated, but the approach success has been limited (Krueger, 1996). The digitalization and controlled delivery of olfactory cues also present new opportunities for sensory substitution training. Nevertheless, the primary issues associated with olfactory cues include their chemical synthesis, user’s fundamental attribution and attention bias to other sensory modalities, and perceptual cross-cultural differences (Spence et al., 2017). In addition, the neural mechanisms behind odor coding, such as odor valence and intensity perception, are not clearly elucidated, what makes it difficult to exploit for controlled clinical and DTx applications (Mainland et al., 2014; Sagar et al., 2023).

#### 3.1.4 Vestibular system

The vestibular system provides multidimensional positional and self-motion information, thanks to inner ear mechanoreceptors responding to vertical, linear, and angular acceleration of the head. Vestibular information is critical in self-representation and embodiment mechanisms (Lopez et al., 2008). The vestibular system interplays with visual system, notably through the vestibular-ocular reflex, allowing to adjust eyes movements in response to motion to keep visual focus. In XR, the incongruency between vestibular cues and visual cues, for instance self-motion, plays a major role in cybersickness. Conversely, providing minimal amount of vestibular feedback through user motion or minimal ambulation can help reduce it and improve SoP during XR experience (Kruijff et al., 2015, 2016).

Furthermore, vestibular feedback and ocular torsion tracking in XR are interesting tools for clinical diagnosis and treatment of visual-vestibular dysfunctions and neurodegenerative diseases. Measurement of ocular torsion and skewing responses in response to vestibular cues changes are possible clinical examination tools to detect aberrant processing of visual information (Wibble and Pansell, 2019), diagnose vestibular dysfunctions and monitor optometric or balance rehabilitative therapies (Cohen, 2013). Vestibular dysfunctions are also associated with multiple neurodegenerative diseases and have a major impact on patients’ mortality and morbidity (Cronin et al., 2017; Kouris et al., 2018). Vestibular rehabilitation has shown positive impact in Parkinson disease patients’ motor control (Rossi-Izquierdo et al., 2009; Basta et al., 2011). XR interfaces with dissociable avatar, direct full body visual feedback and paired stabilometry could be a potent tool for such motor and balance therapies. Visual feedback strategies indeed have significant impact on balance therapy efficiency (Walker et al., 2016; Noh et al., 2019).

#### 3.1.5 Haptics and proprioception

Elementary haptic interactions are frequently included in XR interfaces for feedback—to underlie a cue or an action, or for controlling—through specific fingers gestures. Hand-based techniques are the most common approach to implementing grasping, rotation and manipulating metaphors. Visuo-tactile integration often occurs under highly dynamic conditions requiring constant sensorimotor update, such as dexterity tasks. However, manual control and haptic feedback in most current XR interfaces do not mimic human haptics, but require a new task-specific learning, what can impact the transfer of trained skills to real-life. In surgery simulators and manual motor control recovery interfaces, realistic haptic control and feedback are core to the learning process and the skills transfer. Yet, haptic feedback in XR interfaces mostly covers vibratile input through hand controllers, whose complexity merely addresses that of real-life mechanoreceptors, that integrates temperature, pain, and pressure patterns sensing. Most 3D interaction systems do not support the ability to track the individual fingers (Jerald, 2015). This unnaturalistic use of haptic feedback, including in dexterity training applications, is questioned by multiple groups striving to integrate texture and temperature perception into XR haptic feedback (Kato et al., 2019; Junput et al., 2020; Keef et al., 2020), and allow precise finger and naturalistic grip and push movements through manual controllers (Dorfmuller-Ulhaas and Schmalstieg, 2001; Voigt-Antons et al., 2020).

Incongruent visuo-haptic information dilemmas are often solved in favor of visual information (Farnè et al., 2000; Tsakiris and Haggard, 2005). This might explain the relatively low troubles created by over-simplistic haptic feedback when associated with rich visual content. This mechanism is also exploited in clinical XR interfaces addressing phantom limb syndrome in amputee patients (Hunter, 2003; Foell et al., 2014). However, the visual dominance is less prominent during active haptics and self-generated motor tasks (Tsakiris et al., 2006; Rognini et al., 2013; Boban et al., 2022), what highlights the interference with proprioceptive signals, and the need for realistic feedback solutions for sensorimotor trainings. The implications of visuotactile integration in body ownership and out-of-body experience also suggest potential for further avatar therapy, awareness, and immersion research (Pavani et al., 2000; Rognini et al., 2013).

Proprioception through tendons, muscle spindle and joint mechanoreceptors, is frequently regarded as a distinct secondary haptic system. It provides crucial information for self-body perception, such as muscle tension and joints angles, what informs users on their body angle, and whether their movements are self or passively induced. This information is central to body ownership (Tsakiris et al., 2006; Lopez et al., 2008; Ehrsson, 2012; Butler et al., 2017). Incongruent proprioceptive cues in VR are contributing to cybersickness, lower SoP, and can potentially have a negative impact on motor control rehabilitation processes (Pritchard et al., 2016; Gallagher and Ferrè, 2018; Schlienger et al., 2023). XR interfaces allowing users ambulation and full body kinematics seem a more suitable alternative (Slater et al., 1995), but comparative research still misses. VR interfaces for specific upper limb proprioceptive rehabilitation have been parallelly developed for post-stroke and movement disorders patients (Wong et al., 2012; Abbruzzese et al., 2014), but proprioceptive feedback is not systematically implemented in motor rehabilitation processes. Yet, the occurrence of visuo-proprioceptive integration during joint visual and proprioceptive stimulation and its positive effect on motor learning provides new perspectives for rehabilitation (Wong et al., 2012; Schlienger et al., 2023).

Haptic feedback is also developed as an accessibility feature, for instance to allow deaf audience to feel ambient sounds through vibratile floors or vest (Shibasaki et al., 2016; Hashizume et al., 2018), or to provide information to visually impaired VR users through augmented interactive white cane or braille display (Ghali et al., 2012; Kim, 2020). Such features could ultimately be integrated into occupational therapy practices, but also in XR interfaces for diverse applications and general patient users.

#### 3.1.6 Sensorimotor interactions

Active interaction thinking is critical in XR design. Immersive design does not only comprise 3D visualization of virtual objects but also techniques to interact, manipulate, and gain knowledge from these objects. To interact with the virtual objects in XR, the user is requested to perform real-world actions or symbolic “magical” actions. For non-motor-skill-specific tasks, such as cognitive games or data exploration, sticking to a close real-world-action for every interactive task might lose user’s engagement and slow down its progression and focus. A common trade-off design is to allow magical license for contextual or navigating interactions and focus on realism for perceptual and motor tasks (LaViola et al., 2017). Traditional sensorimotor interaction techniques from 2D interfaces rely on a set of manual simple interactions (pinch, drag, rotate). 3D interactions can also be performed through handles joysticks, what allows simple but limited interaction. Verbal or gaze interaction control have been marginally investigated in 2D (SpecialEffect Studio, 2023). Their extension into 3D has an enormous potential for disabled user accessibility, but also to prioritize the sensory modalities to be trained or investigated. In large MR environments, telerobotic is also explored for 3D interaction, notably to provide personal assistance to medical students during care simulations (Sampsel et al., 2014; Molloy et al., 2016; Gobron, 2020).

Interaction is often limited to rotating the viewpoint, zooming, targeting, or releasing text information, what leads to lack of usability, low quality experience, and limited engagement and training efficacy (LaViola et al., 2017). The best solution implemented to date originates in the development of realistic shooting games: by combining 3D display and 2D interaction designers force users to successfully train 3D cognition, situational probability and anticipation skills, transferable to real-life situations (Green and Bavelier, 2003; Feng et al., 2007; Basak et al., 2008; Zelinski and Reyes, 2009). These benefits were also found to be partially transferable to overall executive function improvement in older adults (Basak et al., 2008), and are exploited as a GfH in geriatric psychology (Stern et al., 2011). In psychiatric applications, the passive exposition to virtual stimuli is thought to be a more central therapeutic component than the interaction itself (Grochowska et al., 2019). However, for pedagogic or rehabilitation applications, this lack of complex and realistic interaction can be a major struggle. Sensory feedback is most lacking. Efforts towards realistic and operable sensory feedback has been made by neurosurgery simulators developers, who notably introduced various force feedback depending on the mechanical properties of the biological tissues and the force exerted by the users (Alaraj et al., 2011, 2013). Though, the majority of currently used interfaces for surgery training only provide visual and rough vibratile haptic feedback (Mao et al., 2021).

### 3.2 Cognition and game design

Working and long-term memories of situation, decisions, actions, and their consequences are essential for interacting and training, either for complex declarative tasks (such as informative educative interfaces), or procedural tasks. Initial immersive exposure and training allow users to acquire situational awareness, i.e., to internalize a cognitive model of themselves within the trained environment. Situational awareness includes time and space awareness, understanding of the spatial relationships, of the other players of the interaction, and possible action outcomes evaluation. Situational immersion is often the only way to acquire awareness of precise sensorimotor actions, such as surgical gestures or fine coordinated motor control: only the physical experience and sensorimotor feedback can build the cognitive and proprioceptive representations necessary for such learning. Conventionally, these skills are typically acquired through experimentation. The closed sensory information-processing feedback loop is crucial, as concomitant motor actions and positive sensory and proprioceptive feedback serve to strengthen action loops at both the associative and local circuit scale (Schouenborg, 2004; Makino et al., 2016). By adding multiple components to this action-perception loop, such as verbal cognition (e.g., explicit rules, knowledge, mention of the goal), the learning process can be declarative, conscious and proprioceptive at the same time, and the user leverage multiple cognitive strategies (McDougle and Taylor, 2019).

From a game design perspective, the initial situational awareness can be promoted by realism, storytelling, and use of user’s real-life priors. XR environments are characterized by a set of rules that define virtual objects’ physics, actions goals and narrative; providing the user with a framework to analyze, anticipate and act with the virtual objects. Rules are the gateway to both XR interaction and serious objectives, as they “set up potential actions, actions that are meaningful inside the game, but meaningless outside” (Juul, 2005).

Game mechanics include abstract mechanics—the physics and probabilistic rules governing the gameplay; and representational mechanics,—the elements that are directly tied to the game context, storytelling, and progression systems. Representational mechanics include features such as visual level of detail, time space, and point of view, which can exert a significant influence on embodiment (Juul, 2005; Romano et al., 2016). In XR-based clinical applications, whether explicitly gamified or not, abstract and narrative representational mechanics are deeply intertwined. One on hand the narrative coherence and the adhesion to the immersive environment is key to skills acquisition. However, the generalization of these skills in real-life settings depends on interaction realism. Abstract game mechanics can be implicit or explicit—through goals declaration, or tutorials; common or game-specific; magical or relying on real-world rules—e.g., gravity, probability of adverse events, emotional faces, trust relationship building. In recreational video or immersive games, users adapt and perform faster when they can apply their real-life priors to the game actions (Johnson and Wiles, 2003; Bodi and Thon, 2020). This prior transfer is considered as a core element of flow (Johnson and Wiles, 2003; Jennett et al., 2008). In GfH for medical education and skills training, medical prior knowledge impacts performance, and vice versa, gaming experience updates students’ priors (Lee et al., 2019; Hofmann et al., 2020). In GfH, real-life physics and social behaviors are crucial for, respectively, motor and communication skills transfer. Nonetheless, XR application focusing on sensorimotor skills acquisition can accommodate fictive magical environments, declarative goals or even magical interaction with secondary objects or controllers. On the opposite, behavioral interventions or psychotherapy applications can tolerate some space, time, or haptic feedback detour, but they should encompass a realistic narrative sequence, realistic characters interaction, body movements and facial expression cues, making realistic visual display inseparable from realistic storytelling.

Today, most GfH and XR-based interfaces used for clinical applications are system-driven games of progression, focused on abstract game mechanics. Compelling storylines and narrative gaming universes, though rarely implemented in clinical applications, have the potential to enhance immersion, long-term engagement, and enactment of the user, that has a meaningful impact on the story (Juul, 2005; Bodi and Thon, 2020). This can be especially important in vulnerable populations. In pediatrics and disabled populations, demotivation and disengagement often lead to discontinuation of therapy, depression, and worsens patient outcome (Zihl, 2010; Zahi et al., 2016; Finn et al., 2018; Levac, 2023). This is especially true in therapeutic domains that require repetitive drills, such as motor, speech, and visual rehabilitation therapies. Narrative design may also be of particular interest for psychotherapeutic and communication applications. On the other hand, games of emergence offer higher cognitive complexity and decision branching trees that are essential to numerous clinical applications, such as clinical education, exploratory learning for motor or sensory rehabilitation, and behavioral interventions. Specifically, exploratory games offer interesting models for puzzle-solving design in the perspective of diagnosing cognitive functions, as well as for preventive and rehabilitative training (LaViola et al., 2017; Chicchi Giglioli et al., 2018). Games of emergence also offer unpredictable challenges and rewards. Nevertheless, it poses significant challenges in terms of reproducibility, standardization, and monitoring when considered for educational and therapeutic applications. The impact of challenge rarity and variability on sensorimotor plasticity in the context of serious games remains unknown. The design mechanics of serious games is a relatively new field that necessitates careful consideration and cross-evaluation with clinical outcomes, particularly in the case of DTx.

Last, evolutive game mechanics can benefit clinical GfH and DTx. Level design strategies are pertinent to create engaging experiences for users, but also provide a framework to monitor their outcomes and assist in clinical decisions. In application fields such as occupational therapy or visual reeducation, therapists play a pivotal role in analyzing clinical progress and level progression. Paired evolutive mechanics and professional monitoring offer opening for better standardized, equitable, and evidence-based level design. It has the capability to incorporate numerous quantitative variables derived from user behavior and history, while reducing therapist implicit biases (Hall et al., 2015; FitzGerald and Hurst, 2017; Backhus et al., 2019; Garb, 2021).

### 3.3 Assessment

The assessment of efficiency and usability of clinical interfaces typically occurs concurrently during a post-production testing phase or clinical trials for DTx. Irrespective of the particular uses, the evaluation of clinical efficiency is assessed through broad functional outcomes, success rate, or memory tasks, while the usability is assessed through questionnaires and eventually medical sociology methodology (Culyba, 2018; Wang et al., 2023). Immersive interfaces could benefit from detailed assessment and analysis of users sensory, behavioral, and cognitive responses during interactions, proper to neurodesign studies (Auernhammer et al., 2023). In tailored applications development, intermediate psychomotor, physiological, and electrophysiological correlates would though be critical evaluation variables (Liu et al., 2017; Chicchi Giglioli et al., 2018; Kim, 2020; Miskowiak et al., 2022; Jiménez-Rodríguez et al., 2023).

In current neurodesign toolkit, eye tracking stands out as the most widely utilized and accessible method for evaluating user behavior and human factors. Usage includes safety studies (Manhartsberger and Zellhofer, 2005; Han et al., 2020), health design validation (Champlin et al., 2014; Erol Barkana and Açık, 2014), and to a lower extend visual communication (King et al., 2019; Kredel et al., 2023) and medical education (Ashraf et al., 2018; Lévêque et al., 2018) research. Visual behavior tracking can provide critical information about attention, spatial orientation strategies, pattern recognition strategies, and visual functions (King et al., 2019; Titchener et al., 2019). However, most design, communication, and usability concentrate on the locations of gaze fixation (Manhartsberger and Zellhofer, 2005). Downscaling gaze patterns analysis is an interesting research opportunity for the advancement of visuo-cognitive analysis in clinical applications.

Sensory assessment and adjustment allow to further link the virtual and real-world environments, closing the loop between real-life perception and virtual action through the provision of real-life sensory consequences. In contemporary MR interfaces, eye tracking and spatial mapping allow to align the virtual actions with real-world perceptual rules, at least on the visual sensory modality. The next generation of interfaces is focused on expanding this mapping to include other sensory modalities. Hand-tracking has been the subject of particular investigation (Dorfmuller-Ulhaas and Schmalstieg, 2001; Xiao et al., 2018; Voigt-Antons et al., 2020). K. Dorfmuller-Ulhaas pioneered the research on detailed optical kinematic hand tracking, which enabled the grasping of objects with natural finger closure movements (Dorfmuller-Ulhaas and Schmalstieg, 2001). The potential to circumvent controllers and naturally interact with objects provides great opportunities for complex surgical training and motor control rehabilitation (Buń et al., 2022). However, the current reported usability does not exceed that of traditional controls (Voigt-Antons et al., 2020).

In DTx, sensory and cognitive responses are the bottom line of the clinical evaluation. Their assessment and design are particularly challenging, as the nervous system interfaced can be impaired at multiple levels—e.g., sensory or motor nerve degeneration, cognitive impairment, attention deficits, circuits that have undergone plastic adaptation to sensory deprivation. It is essential to comprehend the ways in which particular users interact with and utilize virtual content, as well as to identify the specific adverse effects and safety concerns that may arise from immersion, HMDs, visual simulation, and wearable technology used for evaluation in these users. Risks encompass worsening of condition, hallucinations, epilepsy outbreaks, loss of balance, falls and physical injury (Sutcliffe, 2003; Garrett et al., 2018; Tychsen and Thio, 2020). The inclusion of at-risk patients poses an ethical challenge for DTx evaluation: interfaces are either clinically evaluated as a class III device on risky population (Wang et al., 2023), what renders co-design, multiple versions, and iterative design almost impossible, either evaluated based on general usability outcomes. A first framework has been developed by the Spatial Perception and Cognitive Experience (SPACE) Lab design research group. To design visual space for epileptic residents, they conducted preliminary exploratory study was conducted to gather evidence on the perception of various spatial features in their end-user epileptic population, based on their visual and interaction behavior. They used *in-situ* eye tracking during VR sessions, and psychological evaluations in complement to participatory design discussion (Kwon et al., 2023). The singularity of this methodology – that is referred to as Participatory Neurodesign framework, and that of its design outcomes, underscores the current lack of evidence and joined studies interfacing perceptual sciences, design research, and patient populations. Simultaneously, it presents a novel applied neurodesign framework for evidence-based design intended to neurodiverse and patient populations.

### 3.4 Social and ethical design challenges

With the increasing use of XR and gaming in the healthcare sector, there is also an increasing analytical observation of these applications by medical and technology ethicists. Schmitt-Rüth and Simon develop a socio-ethical model to evaluate GfH design process (Schmitt-Rüth and Simon, 2020). Four fields are to be included in such evaluations:

- Safety: the basic need for user safety describes the integrity of their health, physical and psychological well-being. Interface usage should be free from harm, respect privacy and confidentiality.
- Equity and Participation: this evaluation domain captures concepts such as solidarity, fairness, equality and inequality, discrimination, stigma, rights, inclusion and exclusion, accessibility, affordability, ownership, universal access, employment.
- Sustainability: aspects such as efficiency, effectiveness, social sustainability, economic sustainability, environment, profitability, and cost are the focus of this category. The technology impact of the user’s living environment is of particular importance.
- Self-determination: this field evaluates dependence, controllability, and ease of assigns of the developed technology, but also confidentiality, privacy, and data protection.

A sustainable development in the sense of these four fields is achieved by comprehensive stakeholder analyses and “acceptance workshops,” in which the mentioned problem fields are analyzed and considered case-specifically, possibly with end-users’ participation. Arora and Razavian postulate that the overlay of virtual and real norms, and the conflict “between the interests of individuals subjected to gamification and those who provide or design gamification elements” are the two primary reasons for the *prima facie* ethical issues related to gamification (Arora and Razavian, 2021). They therefore propose a model that attributes “[r]esponsibilities for proper design” to designers: designers should facilitate “proper use ensuring proper embedding of the apps within the larger social context.” Designers thus need to share responsibilities with stakeholders such as the public, patients, physicians, biomedical engineers, and health insurers, in order to enhance the outcomes of applications and effectively educate users. Active participation in democratic social discourse and self-reflection by designers regarding the social and economic implications of their technologies are also crucial.

In addition to ethical considerations, the specific nature of XR as an audiovisual, haptic, and interactive experience also raises some very narrow questions. First, virtual embodiment can lead to induce emotional, cognitive, and behavioral changes, intended and unintended, and could ultimately lead users to develop addiction symptoms (Slater et al., 2010b, 2020; Neyret et al., 2020). Owing to the high persuasive power of artificial environments, not all effects are foreseeable (e.g., in the form of unintentional retraumatization); this possibility must also be factored into the ethical and responsibility considerations. Second, long-term vision safety concerns were raised regarding XR therapeutic use, especially in children; however no long-term effect on visual functions were observed (Ha et al., 2016; Turnbull and Phillips, 2017; Tychsen and Foeller, 2020; Iskander et al., 2021). Last, the sensory aspects of interaction design are subject to sociocultural biases: sensory dominance varies with specific population cognition (Feldman et al., 2018; Murray et al., 2018). And the social hierarchy of senses subjective relevance is not universal (Sharma, 2023). Multisensory design and evaluation should therefore consider the cultural aspects of the user population. At present, the clinical testing of XR-based DTx does not include multinational clinical trials, and very few trials were held in Asia to date (Wang et al., 2023). Extending the tested population of clinical users would enable the identification and mitigation of cultural biases in UX design (Slater et al., 2020; Oliva et al., 2022).

The ethical consideration of accessibility is a significant issue in contemporary XR interfaces. XR interfaces include specific accessibility features when they address specifically impaired populations in a rehabilitation perspective—but limited to the specific sensory modality addressed. The vast majority of educational, exploratory, and informative technologies lack general accessibility features (Wang et al., 2023). Including and balancing accessibility in XR is a major challenge, mostly originating from hardware settings, controllers design and sensory feedback design. Quite interestingly, these challenges technically overlap with the need for multisensorial modalities integration. Indeed, a major principle of inclusive design is to provide redundant sources of control and information, so that diverse sensory modalities can be used in alternation or in custom combinations (Dudley et al., 2023). The generalization of inclusive design effort has tremendous benefit potential to both abled and disabled user population: allowing social, professional, and medical inclusion to the former, and enhancing immersive experience and clinical outcomes in the latter. This design aspect could therefore be a relevant indicator that XR technologies are achieving maturity and are ready to take root in educational and clinical settings.

One challenge researchers face is accessing enough users with specific disabilities. One potential solution is to collaborate with organizations representing disabled users and to leverage the participation of a diverse audience for web-based platforms. Such user-centric approach is increasingly adopted in healthcare design, predominantly in the development of digital health tools intended to assist clinical staff documentation (Mummah et al., 2016; Marwaha et al., 2022). However, the validation of DTx and diagnosis technologies is based on clinical trials outcomes and evidence cross-validation: this methodology typically excludes iterative processes and the involvement of multiple stakeholders, or limits their involvement to the preliminary conception phases.

### 3.5 Clinical status challenge

The development of home-based digital rehabilitation interfaces raises some concerns. XR interfaces can be seen as potential solutions for addressing the shortage of clinical staff and medical isolation (Khan et al., 2023). Importantly, current immersive DTx are not intended to substitute clinical therapists. XR interfaces can only offer a facilitated environment for patient-therapist interaction, and for exercises repetitions. In physical and sensory rehabilitation, synchronous validation and monitoring of exercises by a reeducation specialist are crucial, as poorly executed exercises are a major threat to therapy efficacy. Finally, human care relations are central to patient engagement, commitment, and long-term therapeutical outcomes (Beach et al., 2006). The biopsychosocial (BPS) care model postulates that multiple levels participate to disease, healing and rehabilitation, and that these multiple levels—physiological to social—must be addressed together in the clinical practice (Engel, 1977; Adler, 2009). BPS model strongly influenced medical education and nursing practices over the past 50 years: XR simulation-based nursing education can be seen as a direct heritage of this framework. However, the integration of digital health in BPS and relationship-centered care is still to be defined.

DTx products have been approved and commercialized for evidence-based therapeutic interventions in the US since 1999 and in Europe since the early 2010s (US Department of Health and Human Services, 1999; Hong et al., 2021; Wang et al., 2023). However, their implementation into current clinical practices is limited by insufficient efficacy evidence, and lack of appropriate control groups and comparative studies. The absence of regulatory post-approval studies contributes to this information gap. A recent systematic review underlined the notorious absence of multinational clinical trials; the sparsity of clinical trials held in Asia; and the challenges to design blind conditions (Wang et al., 2023). The legal framework and practical availability of DTx greatly vary significantly from one country to another, posing risks of exacerbating care inequalities (European Federation of Pharmaceutical Industries and Associations, 2023). Furthermore, the utilization of DTx interfaces requires prior basic digital education, openness, and some level of cognitive ability, what is a barrier for elderly, isolated, or cognitively impaired patient populations.

## 4 Discussion

XR interfaces have numerous technical potentials to train, assist and develop clinical practice. Its sensory dimensionality reduction promotes attentional focus and targeted learning; it offers explicit and exploratory learning overlay; it can offer multisensory control for accessibility, tracking and closed-loop feedback; it beneficiates from technological and theoretical progresses from entertainment, that facilitates immersion, embodiment, and provide insights for successful gamification. However, the benefits of most of these aspects for clinical outcomes are still unverified. Available studies so far demonstrated that specific XR technologies could teach sensorimotor skills, social skills, and declarative knowledge; that they were well accepted by clinicians, health stakeholders and patients; and that recourse to VR could enhance user motivation and engagement. But in numerous sensorimotor trainings or DTx applications, there is no evidence that XR technologies outperform traditional training methods. Multiple XR applications accommodate the traditional role-play, information navigation or physical therapy methods to the digital world and explore their outcomes, rather than taking advantage of new digital learning mechanisms. This exploratory method is complexified by the time and resources needed for both design and evaluation steps. As a result, XR design choices are more often retrospectively evaluated as a whole, rather than subjects to evidence-based iterative processes. The current limitations of XR interfaces for clinical applications likely originate in this retrospective and generalist assessment of efficiency and usability. An approach involving evidence-based design of the multiple interaction mechanics is crucial to align interface functionality with therapeutic or learning goals. Co-design and participative framework can help designers focus on and assess application-specific parameters. There is also room in the current framework for a better integration of the fundamental mechanisms of nervous system plasticity and learning, at the core of clinical training and rehabilitation processes.

A central problem to a comprehensive implementation is the multiplicity and collide of disciplines involved in XR technologies development and medical integration. Only a few studies deal with the social construction of digital games as a new medium in the field of the social system of medicine. The new category “DTx” was introduced for the FDA approval of the game EndeavorRx as a “medical device” for behavioral intervention (Hooker and Karnes, 2022). This discursive shift classified games no longer as entertainment, but as instruments, and only in this way could they be strategically positioned for economic, medical, and governmental stakeholders (Hooker and Karnes, 2022). This positioning requires a second level of multidisciplinary collaboration. In the realm of game production, medical researchers should “be engaged before serious games for health are developed in order to place serious games for health in the best position to have a measurable impact on health outcomes” (Kato, 2013). On the other hand, it is the developers who bring the aesthetic and technological competence to GfH construction, and “are very user-centric and tended to focus almost equally on the problem and the solution spaces when approaching game design” (Cheng et al., 2016). Moreover, many of current XR projects are “driven by game designers and developers, for whom creating a new game is their area of expertise, their comfort zone. When immersed into a healthcare setting, other factors come into play, such as testing, validation, patient-centered outcomes, and evidence-based practice; but how well equipped are the gaming and healthcare professionals to recognize the underlying nature of each other’s field?” (Gendle, 2012). Transdisciplinary knowledge transfer on scientific methodologies is key to uncovering the potential of currently developed XR technologies.

The testing and validation of game mechanisms and technology are the cornerstones of this intertwining. There are few windows in the traditional game development pipeline that allow detailed clinical evaluation and behavioral feedback. White box stage and pre-production playtest may allow designers to validate level design, representational mechanics, and identify specific features needs. Yet, for medical applications, both the clinical approach and its concrete implementation influence its efficacy. An early white-box validation of game mechanics could guarantee the relevance of the VE and framework for the clinical approach and learning mechanisms; while a late evaluation of polished displays and interactions can address realism, immersion, and technical accuracy. However, for DTx, playtesting and redesign iterations are limited by time and resources and design features are often subjectively driven by designers. Iterative clinical evaluation is usually not possible, because robust intervention studies require blinding to prevent bias. This includes avoiding the introduction of bias by engaging users in favor of the intervention asking them active feedback (Birckhead et al., 2019; Chidambaram and Josephson, 2019). In DTx, the clinical efficacy can only be evaluated with a beta version, which is already tremendously late in the development process. It also raises experimental evaluation problems: which game features are participating in the clinical outcome, and need to be evaluated as clinical variables? No study to our knowledge has dissected game mechanics into clinical variables. Another major problem of testing clinical outcomes during playtesting is that user transformation—sensorimotor skills acquisition, perceptual learning, and plastic changes in the nervous system—requires extensive amount of time and repetition. In GfH testing, the solution adopted is to focus on user engagement, and discard clinical evolution. Transformational success in GfH is often measured through game progression, suggesting that completion of the game leads to transformation (Culyba, 2018). However, completion of the tasks itself tells nothing of the retention, generalization of the acquired knowledge and skills.

Solutions are needed to conciliate the requirements, expertise, and procedures from both worlds. Assessment plans with mixed methodologies—including sensory, behavioral, and cognitive neuroscience tools, together with ethical considerations and participatory evaluation windows should be considered. Participatory frameworks offer a base to incorporate behavioral and physiological evaluation in both the co-design and post-design phases. In particular, Participatory Neurodesign (PND) framework represents an initial effort to facilitate the convergence of disciplines (Kwon et al., 2023). PND framework originates in applied research from built environment and wayfinding studies (Edelstein and Macagno, 2012; Rohra et al., 2021; Othman et al., 2023; Dornelese, 2024). In these fields, designers have regular recourse to user involvement and participatory methods, but new challenges arise when wayfinding or environment are designed for healthcare practices and users with specific medical and/or cognitive needs. The healthcare design field is in the search for more evidence-based methodologies, notably for domains peripheral to care, such as built environment, management of care and therapy, occupational therapy, digital health, and among it XR applications. This research accelerated the development of neurodesign as a discipline bridging cognitive sciences with UX. Design of XR interfaces able to exploit complex learning mechanisms require this multidisciplinary consideration. Indeed, while XR systems inherently simplify sensory experiences due to their reductionist nature, this does not confine them to a purely behaviorist perspective in their transformative action on the user. We have discussed earlier how our understanding of interactions and learning mechanisms is bounded to disciplines and scales, and the risks of reductionist or metaphorical perspectives alones. PND framework is one way to combine reductionist validation—intrinsic to sensorimotor training and necessary to optimize interactions, cognitive and experiential validation—intrinsic to XR media and necessary to immersive learning, and user involvement—necessary for integration of the media in the healthcare or health education practices. It opens up the design process, ensuring that the technologies developed reflect both the clinical and experiential needs of the users. This is especially valuable in healthcare, where new digital technologies often face resistance from both patients and professionals due to their integration challenges and the complexity of demonstrating immediate, critical improvements. Participatory design is key to address these acceptation and integration challenges. In GfH development, participatory design improved the effectiveness of complex games, notably when users where involved in game dynamics, levels, and game challenge design (DeSmet et al., 2016). Integrating a data-driven neuroscience component into participatory design further connects this discussion with formal clinical metrics. This is essential to balance the usability and the effectiveness of the technology; whose ultimate objective is a positive patient clinical outcome—either direct or indirect. Open metrics integration also empowers users in discussions with designers, and later facilitates conversations between patients and health professionals. This aligns with the view that health data serve as a medium for care dialogue, and reinforces the place of the patient at the center of their own care journey.
